# Supplementary figures and images for: mTOR signaling regulates demand-adapted hematopoiesis and metabolic reprogramming required for an effective cellular immune response in Drosophila melanogaster larvae
Source: PLoS Genet. 2026 Mar 24;22(3):e1012094. doi: 10.1371/journal.pgen.1012094 (PMC13038113; doi:10.1371/journal.pgen.1012094)

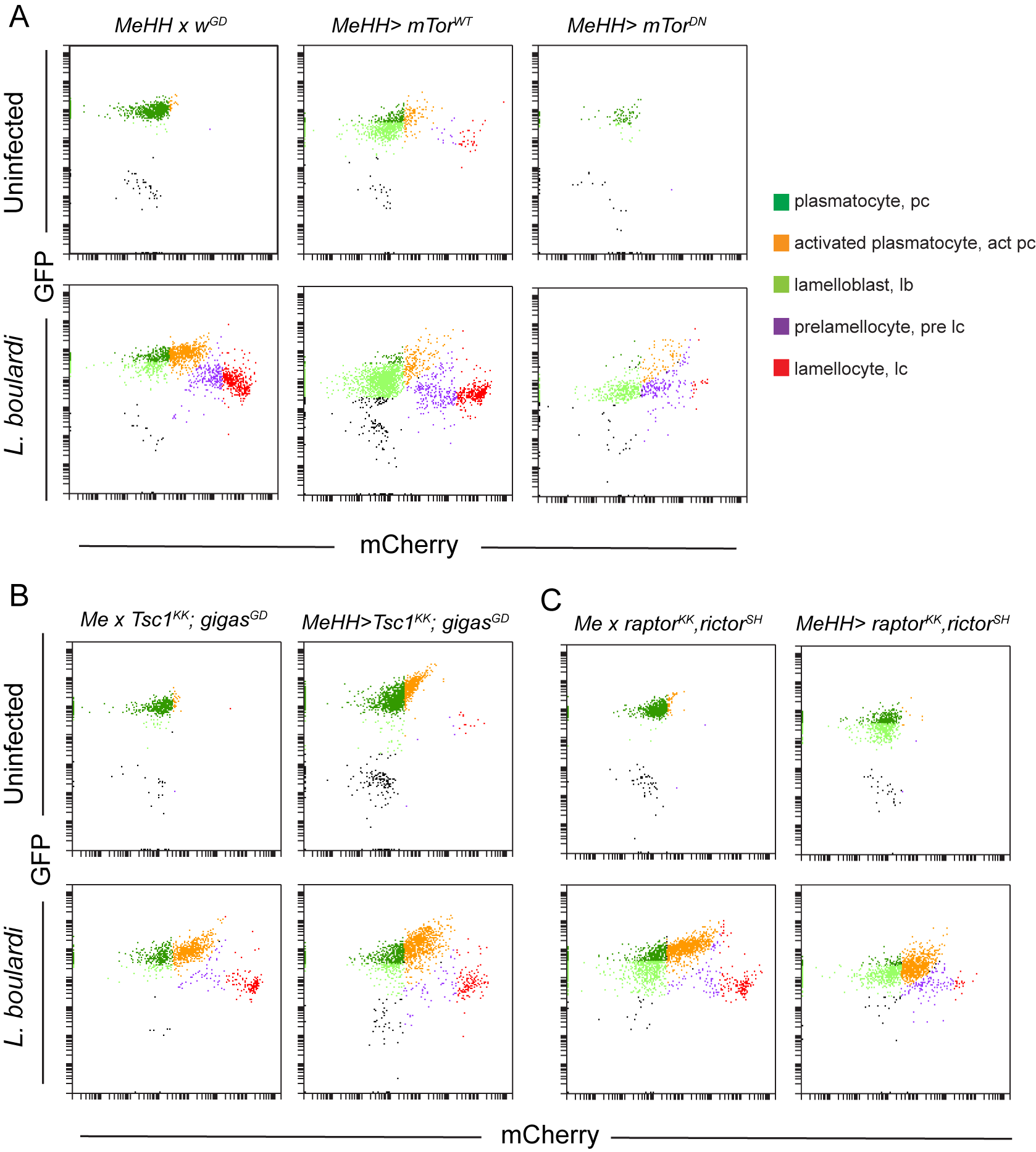

Supplement: S1 Fig — A) MeHH > wGD, MeHH > mTorWT and MeHH > mTorDN (Fig 1). B) Me > Tsc1KK110811; gigGD6313, MeHH > Tsc1KK110811; gigGD6313 (Fig 2). C) Me x raptorKK106491,rictorSH33079, MeHH x raptorKK106491,rictorSH33079 (Fig 3). (TIF) [file pgen.1012094.s008.tif]

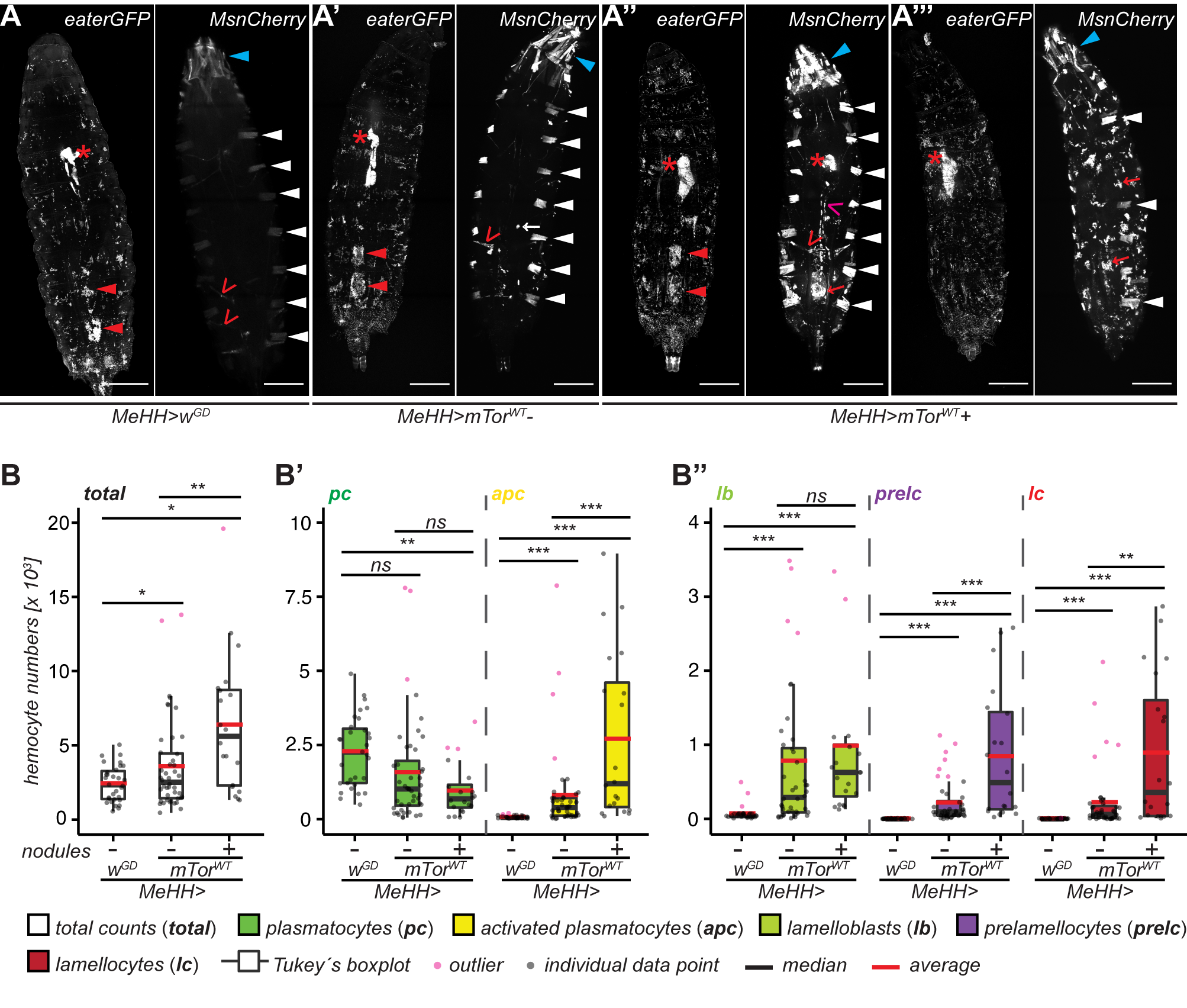

Supplement: S2 Fig — A) In vivo hemocyte phenotype of control (MeHH > wGD), eaterGFP representing the plasmatocyte lineage and MsnCherry the lamellocyte lineage; A’) in vivo hemocyte phenotype of mTorWT hemocyte-directed overexpression larvae without nodules (MeHH > mTorWT-); A”-A”’) in vivo hemocyte phenotypes of mTorWT hemocyte-directed overexpression larvae with nodules (MeHH > mTorWT+). Red asterisk – lymph glands, red arrowheads – segmental peripheral plasmatocytes, white arrowheads – segmental muscles, blue arrowheads – pharyngeal muscles, red V – alary muscle fibres, white arrow – lamellocyte, magenta V – pericardial cells, red arrow – nodule, scale bars 500 μm. B) Total hemocyte counts of control (MeHH > wGD; n = 31) and mTorWT overexpression in hemocytes (without nodules MeHH > mTorWT-; n = 44, with nodules MeHH > mTorWT + ; n = 20); B’) Cell counts of plasmatocyte lineage; B”) Cell counts of lamellocyte lineage. To facilitate plotting, we removed the highest outlier for MeHH > mTorWT+ (5950 lamelloblasts). The data presented in B-B” is identical with Fig 1D-D” but separately plotted for larvae with and without nodules. Significance levels: *** p < 0.0001, ** p < 0.001, * p < 0.05, ns – not significant. The hemocyte count data are available in the S7 Table. (TIF) [file pgen.1012094.s009.tif]

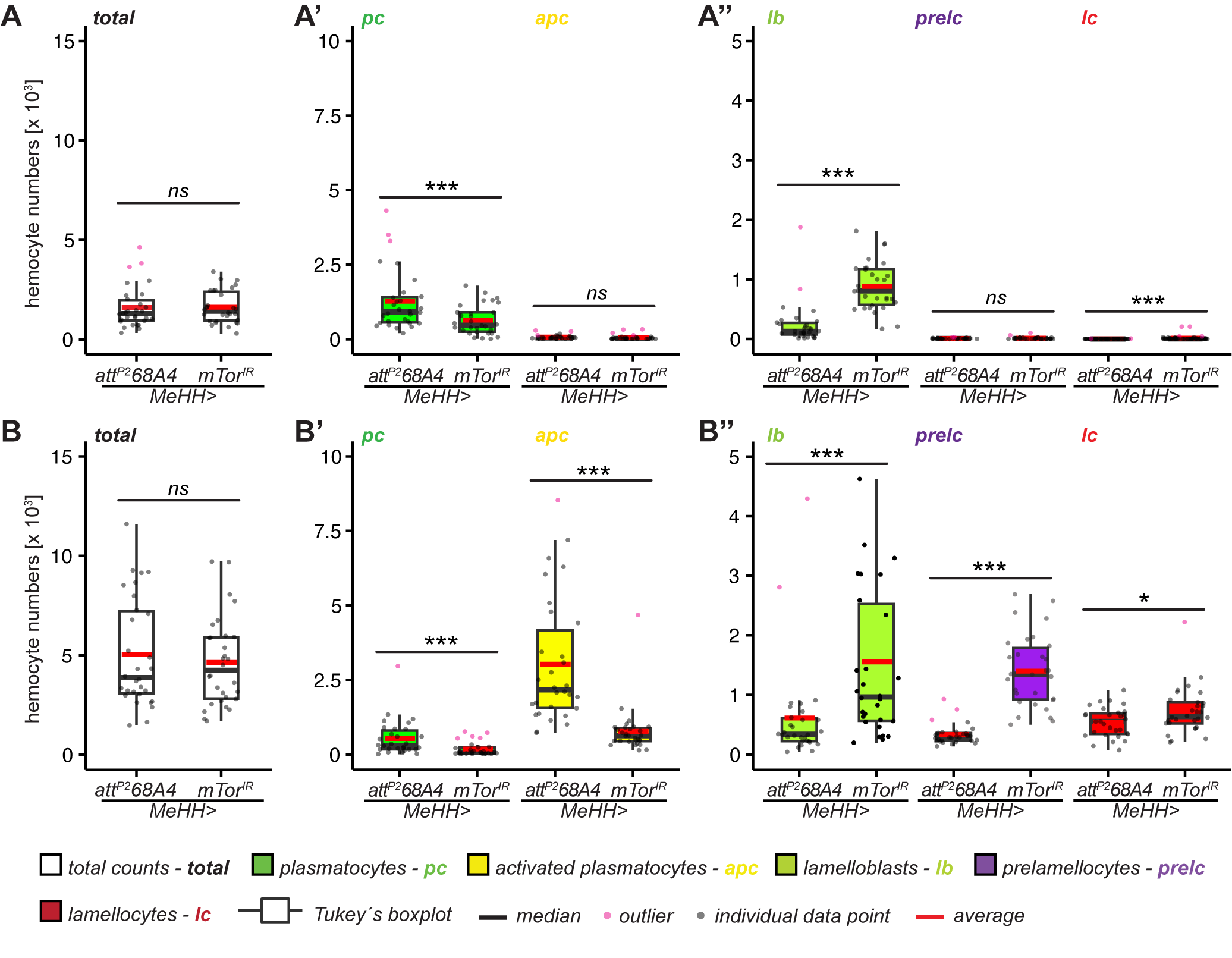

Supplement: S3 Fig — A) Total hemocyte counts of uninfected control (MeHH > attP2684A, n = 30) and mTor RNAi (MeHH > mTorIR, n = 30); A’) Cell counts of plasmatocyte lineage; A”) Cell counts of lamellocyte lineage. B) Total hemocyte counts of infected control (MeHH > attP2684A, n = 30) and mTor RNAi (MeHH > mTorIR, n = 30); B’) Cell counts of plasmatocyte lineage; B”) Cell counts of lamellocyte lineage. Significance levels: *** p < 0.0001, ** p < 0.001, * p < 0.05, ns – not significant. The hemocyte count data are available in the S7 Table. (TIF) [file pgen.1012094.s010.tif]

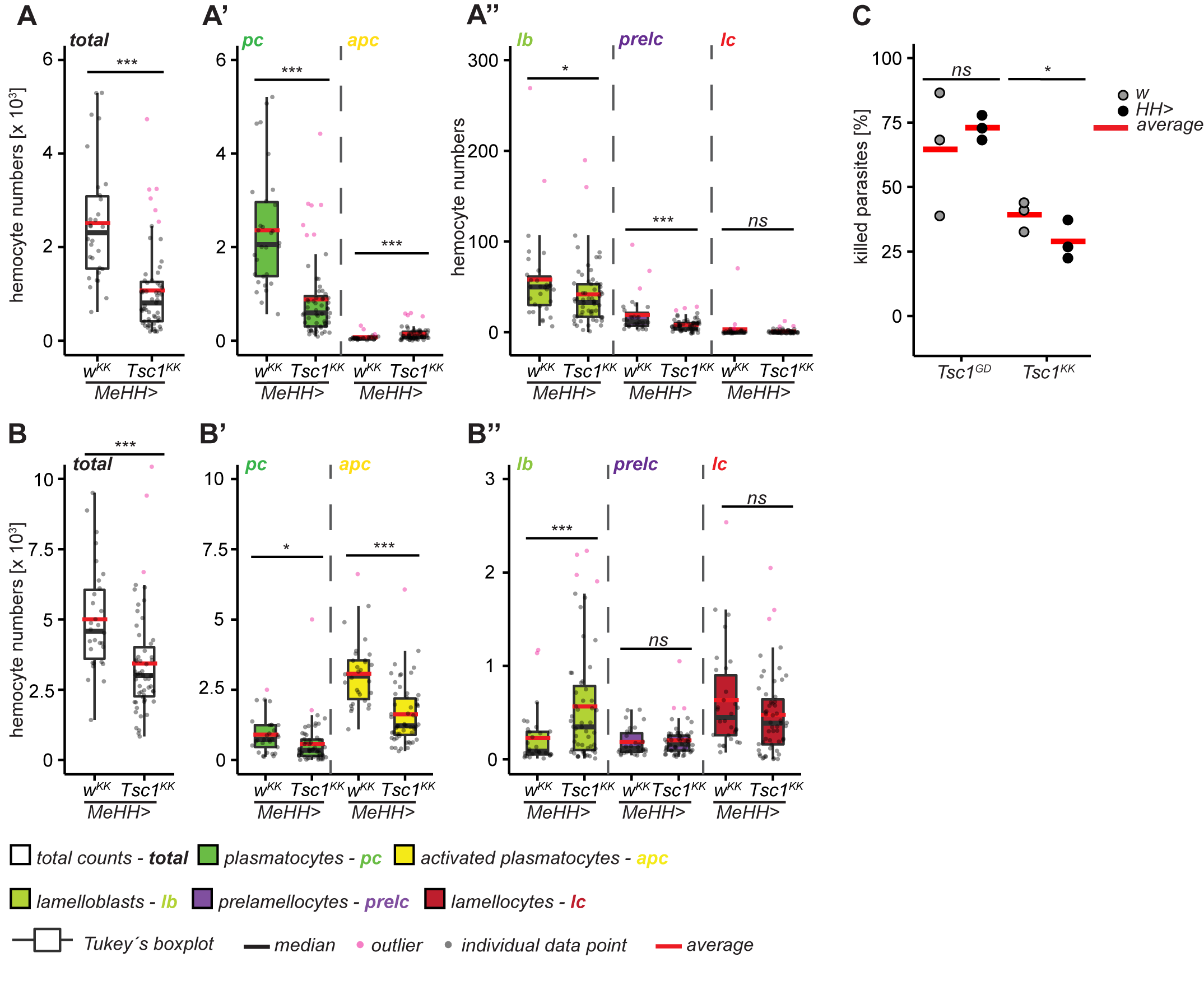

Supplement: S4 Fig — A) Total hemocyte counts (n = 30) of uninfected control (MeHH > wKK, n = 30) and Tsc1 RNAi larvae (MeHH > Tsc1KK, n = 59); A’) Cell counts of plasmatocyte and A”) lamellocyte lineage. B) Total counts of infected control (MeHH > wKK, n = 30) and Tsc1 RNAi larvae (MeHH > Tsc1KK, n = 55); B’) Cell counts of plasmatocyte and B”) lamellocyte lineage. C) Tsc1 RNAi (HH > Tsc1GD, n = 316; HH > Tsc1KK, n = 301) and controls (w x Tsc1GD, n = 316; w x Tsc1KK, n = 301). Significance levels: *** p < 0.0001, ** p < 0.001, * p < 0.05, ns – not significant. The hemocyte count and wasp encapsulation data are available in the S7 Table. (TIF) [file pgen.1012094.s011.tif]

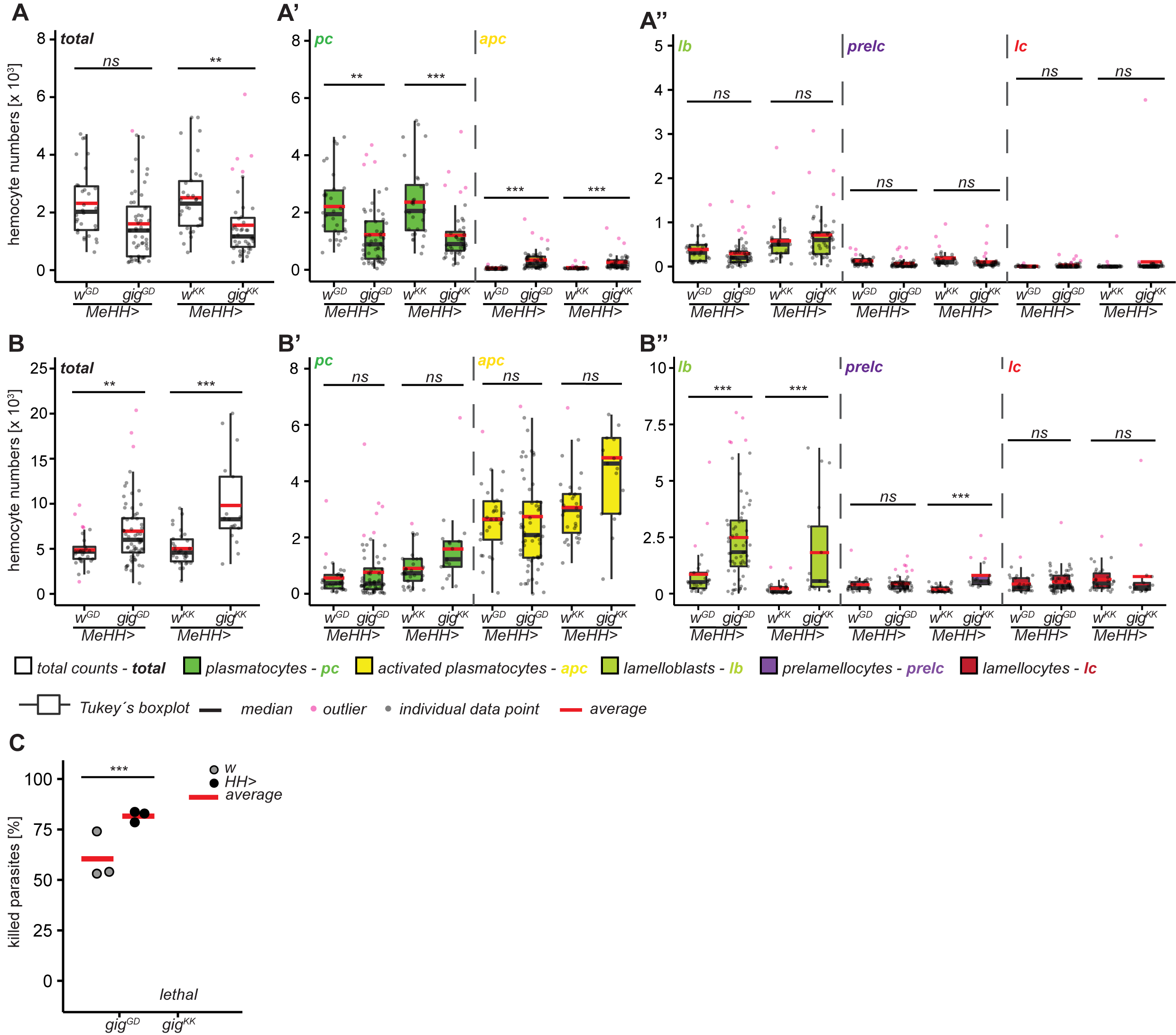

Supplement: S5 Fig — A) Total hemocyte counts of uninfected control (MeHH > wGD, MeHH > wKK, n = 30) and gig RNAi larvae (MeHH > gigGD, n = 52; MeHH > gigKK, n = 43); A’) Cell counts of plasmatocyte lineage; A”) Cell counts of lamellocyte lineage. B) Total counts of wasp infected control (MeHH > wGD, MeHH > wKK,n = 30) and gig RNAi larvae (MeHH > gigGD, n = 61; MeHH > gigKK, n = 17); B’) Cell counts of plasmatocyte lineage; B”) Cell counts of lamellocyte lineage. C) Parasitoid wasp assay of control (w x gigGD, n = 154) and gig RNAi larvae (HH > gigGD, n = 152; HH > gigKK was lethal). Significance levels: *** p < 0.0001, ** p < 0.001, * p < 0.05, ns – not significant. The hemocyte count and wasp encapsulation data are available in the S7 Table. (TIF) [file pgen.1012094.s012.tif]

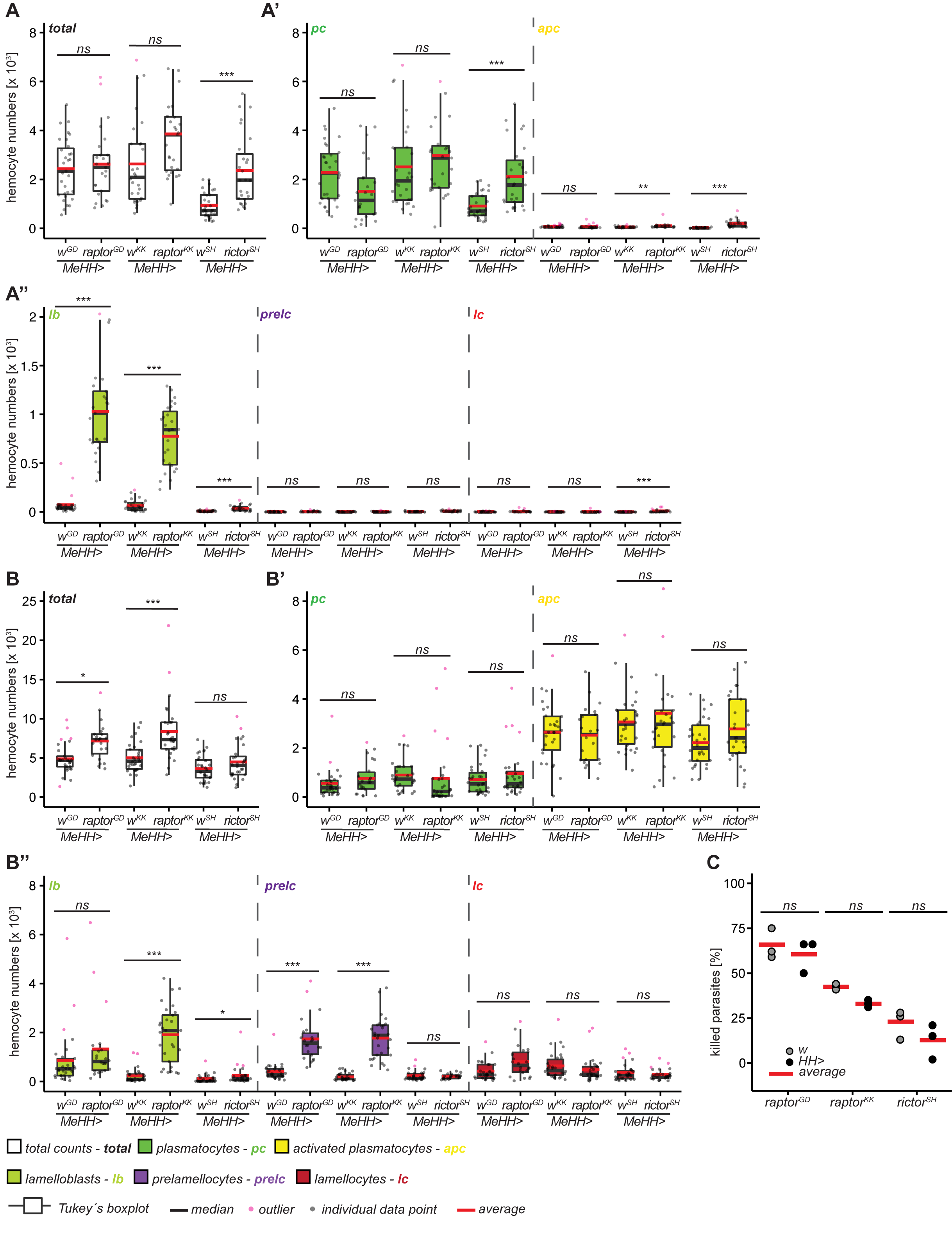

Supplement: S6 Fig — Neither raptor nor rictor RNAi alone block lamellocyte hematopoiesis nor the immune response to parasitoid wasp infection. A) Total hemocyte counts (n = 25–31) of uninfected controls (MeHH > wGD, MeHH > wKK, MeHH > wSH), raptor (MeHH>raptorGD, MeHH>raptorKK) and rictor (MeHH>rictorSH) RNAi; A’) Cell counts of plasmatocyte; A”) Cell counts of lamellocyte lineage. B) Total hemocyte counts (n = 22–30) of infected controls (MeHH > wGD, MeHH > wKK, MeHH > wSH), raptor (MeHH>raptorGD, MeHH>raptorKK) and rictor (MeHH>rictorSH) RNAi; B’) Cell counts of plasmatocyte; B”) Cell counts of lamellocyte lineage. C) Encapsulation assay of raptor (HH>raptorGD, n = 306; HH>raptorKK, n = 301) and rictor RNAi (HH>rictorSH, n = 158) and their respective controls (w x raptorGD, n = 319; w x raptorKK, n = 302; w x rictorSH, n = 141) in hemocytes. Significance levels: *** p < 0.0001, ** p < 0.001, * p < 0.05, ns – not significant. The hemocyte count and wasp encapsulation data are available in the S7 Table. (TIF) [file pgen.1012094.s013.tif]

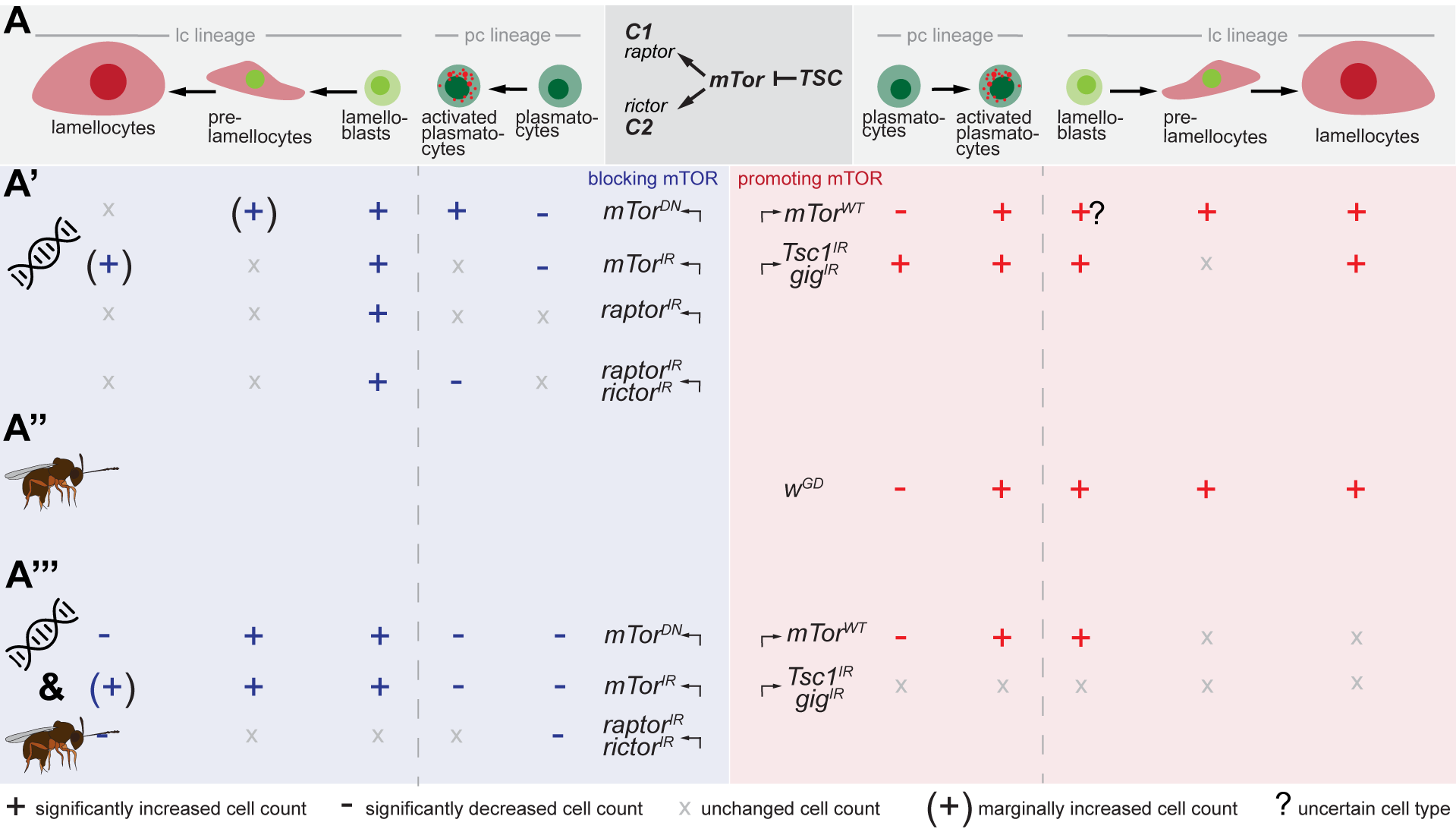

Supplement: S7 Fig — A) Hemocyte lineages (plasmatocyte lineage: plasmatocytes, activated plasmatocytes; lamellocyte lineage: lamelloblasts, prelamellocytes, lamellocytes), mTORC1 (raptor), mTORC2 (rictor) and TSC (Tsc1, gig); A’) hemocyte-directed expression of mTorDN,mTorIR, raptorIR and raptorIR,rictorIR leads to blocking of mTOR signaling (blue) and, in general, to increase in lamelloblasts (marked with “+”) with minor effects on other hemocytes types, while hemocyte-directed expression of mTorWT and Tsc1IR,gigIR leads to the activation of mTOR signaling (red), and to activation of hemocytes including lamellocyte differentiation; A”) wasp-infection in wild type larvae (wGD) reduces plasmatocytes and induces the formation of lamelloblasts, activated plasmatocytes, prelamellocytes and lamellocytes; A”’) expression of mTorDN, mTorIR, raptorIR and raptorIR,rictorIR in hemocytes of wasp-infected larvae reduces the plasmatocyte lineage hemocytes, and in case of mTorDN and mTorIR also increases lamelloblast and prelamellocyte numbers. Expression of mTorWT and Tsc1IR,gigIR have only minor effects in addition to wasp infection. The hemocyte schematics are adapted from Anderl et al. [33]. (TIF) [file pgen.1012094.s014.tif]

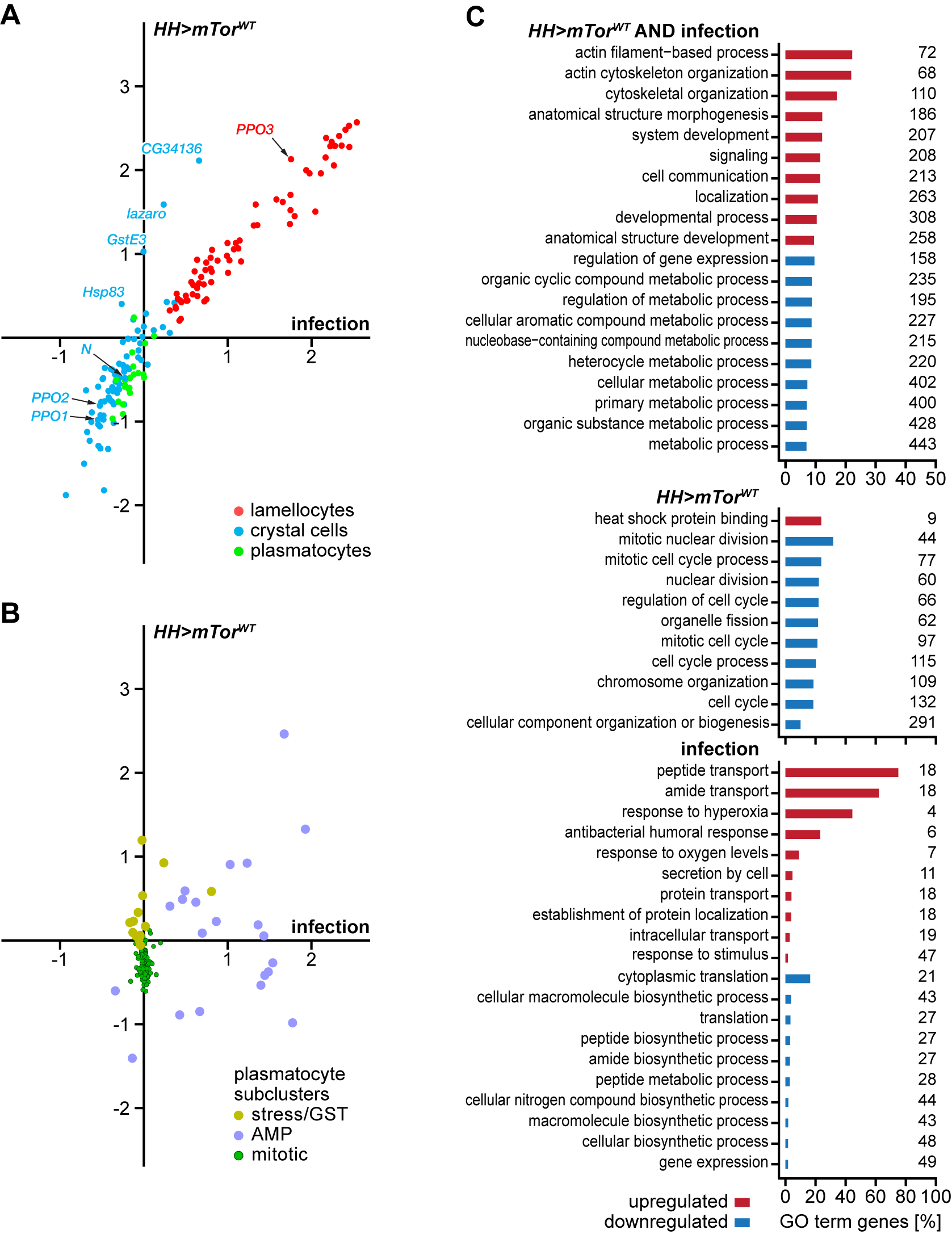

Supplement: S8 Fig — A) Consensus markers for lamellocytes, crystal cells and plasmatocytes. Unlike the other crystal cell markers, Heat shock protein 83 (Hsp83), Glutathione S transferase E3 (GstE), lazaro (laza) and CG34136 were upregulated in mTorWT hemocytes. Notch (N) was downregulated by wasp infection and mTorWT expression. As crystal cell markers, Prophenoloxidase 1 (PPO1) and Prophenoloxidase 2 (PPO2) were downregulated, while the lamellocyte-specific Prophenoloxidase 3 (PPO3) was upregulated by both treatments. B) Markers for selected plasmatocyte subclusters. The “stress/GST” markers were identified both in the clusters PM5 [35] and GST [36], the “mitotic” markers were enriched in PL-prolif [34] and X [39], and the “AMP” genes in at least two of the clusters PL-AMP [34] PM7 [35], AMP [38] and PH6 [39], respectively. C) Representative GO terms enriched in the three groups of genes that are highlighted as green, blue, or red in B. Gene expression data and hemocyte markers are available in the S1 Table and the GO terms in the S2 Table. (TIF) [file pgen.1012094.s015.tif]

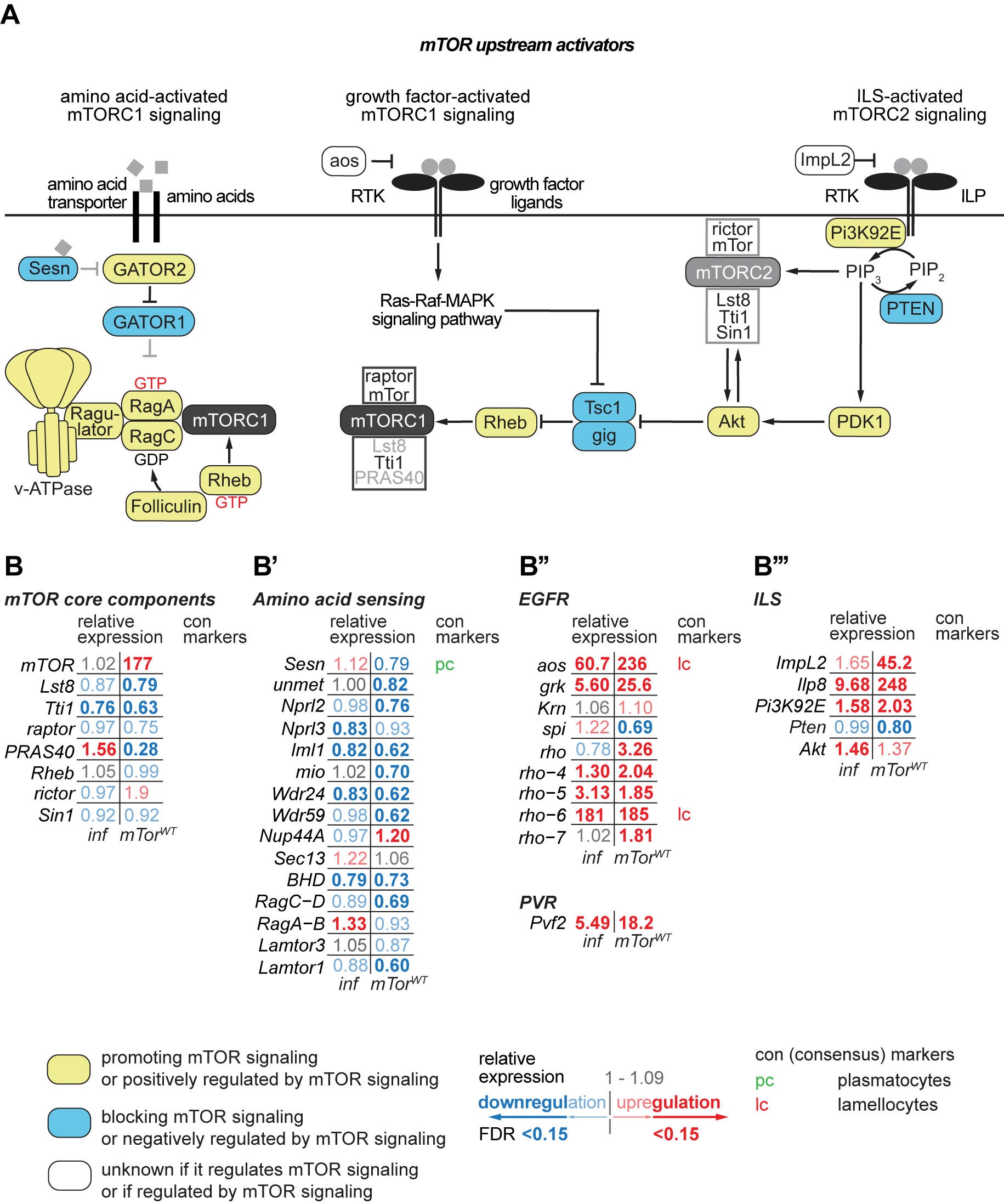

Supplement: S9 Fig — A) Schematic of mTOR complexes and mTOR activating pathways such as amino acid-activated and growth factor-activated mTORC1 signaling as well as Insulin-like signaling (ILS)-activated mTORC2 signaling. B) Expression patterns of mTORC1 (mTor, raptor, Telo2 interacting protein 1 (Tti1), Lst8, Proline-rich Akt substrate 40 kDa (PRAS40)) and C2 components (mTor, rictor, Tti1, Lst8, SAPK-interacting protein 1 (Sin1)). Experimental data in Drosophila suggests that Lst8 is not part of mTORC1, but only of mTORC2 [143]. PRAS40 is thought to affect mTOR activity only in ovaries [144] but is expressed in hemocytes (S1 Table); B’) Expression patterns of genes involved in amino acid sensing. The D. melanogaster amino acid sensors comprise Sestrin (Sesn), which senses methionine, leucine and other branched chain amino acids [145,146], and the S-adenosylmethionine sensor Unmet expectations (unmet; [147]). By binding to their cognate amino acids, the sensors release their inhibition on the GATOR2 complex (grey inhibitory symbol). GATOR2 (Missing oocyte (mio), WD repeat domain 24 (Wdr24), WD repeat domain 59 (Wdr59), Nucleoporin at 44A (Nup44A), Secretory 13 (Sec13)) sequesters and thereby blocks the inhibitory function of GATOR1 (Nitrogen permease regulator-like 2 Nprl2, Nitrogen permease regulator-like 3 Nprl3, Increased minichromosome loss 1 Iml1) on the Ragulator Complex (grey inhibitor symbol, RagA-B, RagC-D and Ragulator (Late endosomal/lysosomal adaptor, MAPK and MTOR activator 1 (Lamtor1), Late endosomal/lysosomal adaptor, MAPK and MTOR activator 3 (Lamtor3)). The D. melanogaster homolog of Folliculin, Birt-Hogg-Dube (BHD) is involved in leucine sensing [148]; B”) Expression patterns of a growth factor antagonist (argos (aos)), of growth factors (gurken (grk), Keren (Krn), spitz (spi), Pvf2) and growth factor processing enzymes (Rhomboid intramembrane proteases (rho, rho 4–7)). The associated RTKs EGFR and Pvr were not differentially expressed; B”’) Expression patterns of In [file pgen.1012094.s016.tif]

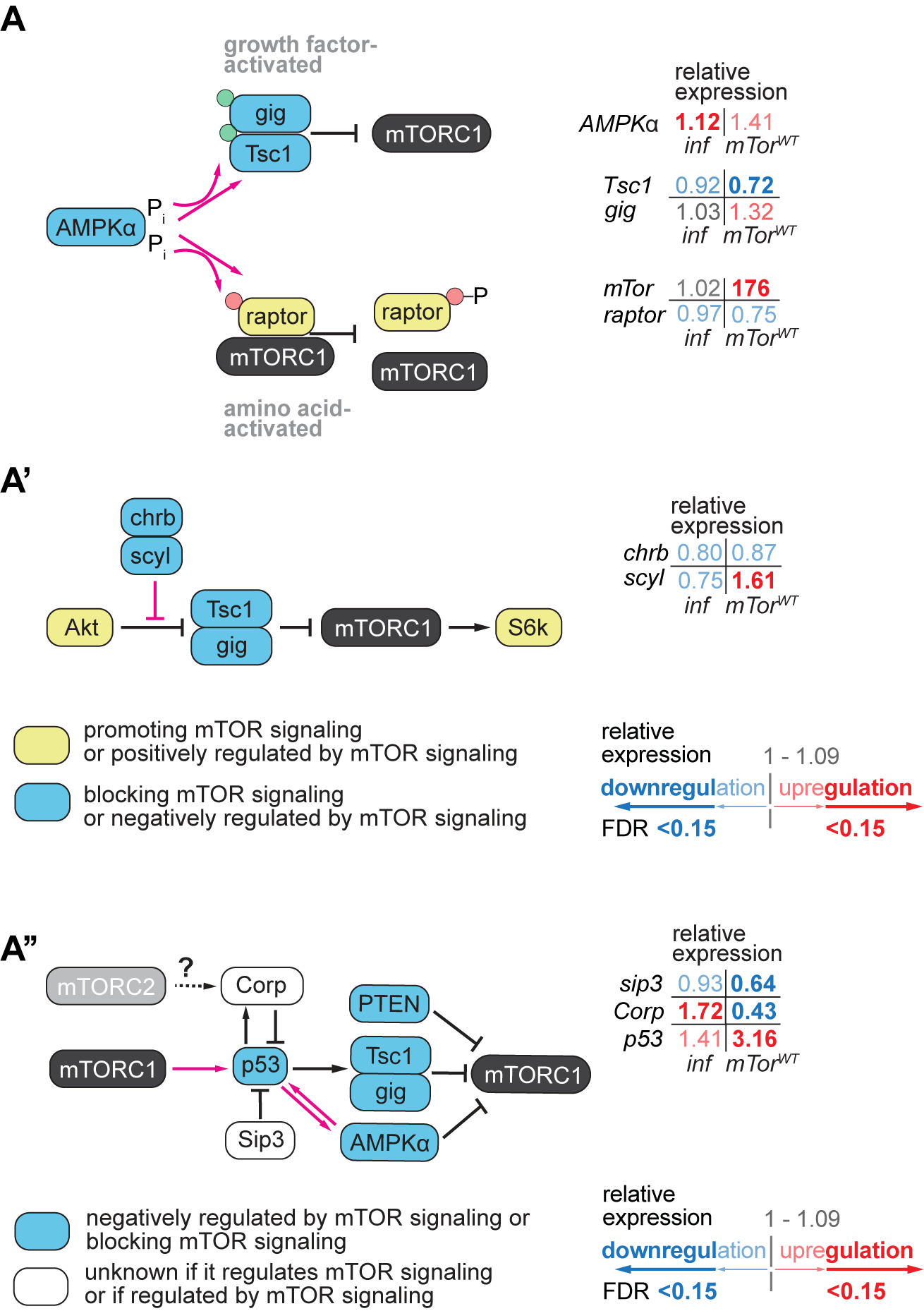

Supplement: S10 Fig — A) Schematic of AMPK-mediated suppression of mTORC1 and gene expression patterns of involved genes; A’) Schematic REDD1-mediated repression of mTORC1 and gene expression patterns of scyl and charybde (chrb); A”) Schematic of p53-mediated suppression of mTORC1 and gene expression patterns of septin interacting protein 3 (sip3), Companion of reaper (Corp) and p53. The gene expression data are available in S1 Table. (TIF) [file pgen.1012094.s017.tif]

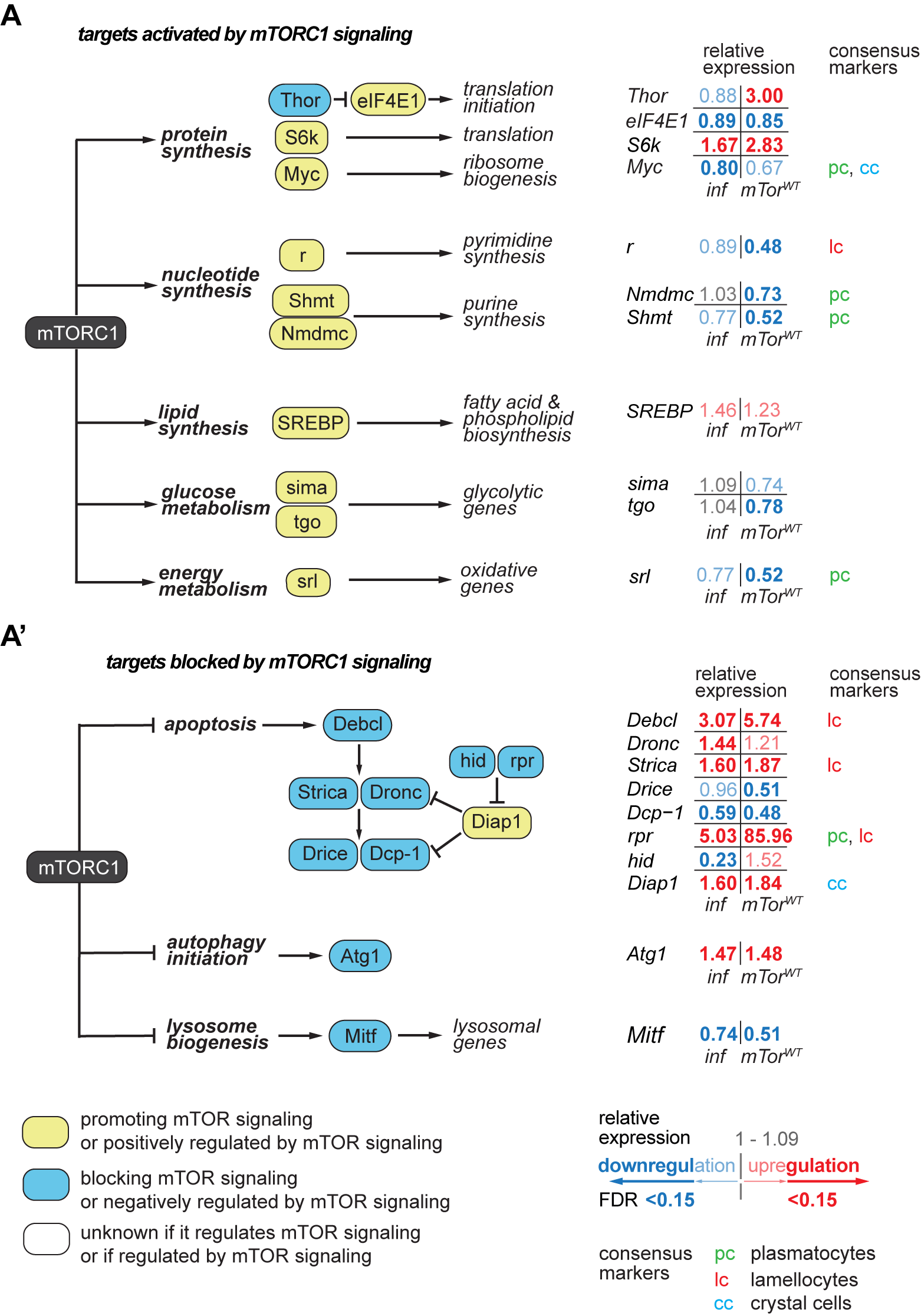

Supplement: S11 Fig — A) Schematic mTORC1-mediated phosphorylation of transcription factors activating protein, nucleotide and lipid synthesis, glucose and energy metabolism as well as gene expression patterns of transcription factors and effector genes in these processes; A’) Schematic of mTORC1-mediated repression of apoptosis, autophagy and lysosome biogenesis as well as gene expression patterns of key genes of these processes. Consensus markers – marker genes for main hemocytes classes based on Hultmark & Ando, 2022 [29]. The gene expression data are available in S1 Table. (TIF) [file pgen.1012094.s018.tif]

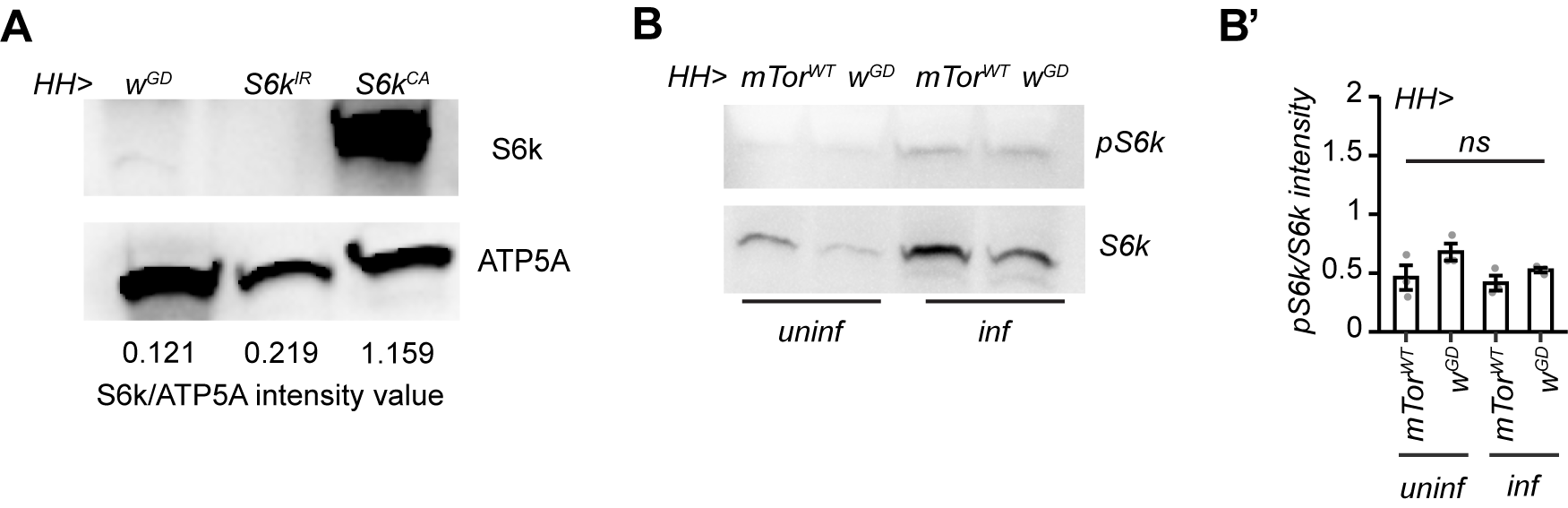

Supplement: S12 Fig — A) To test the newly generated total S6k antibody, we used S6k knockdown hemocytes (HH > S6kGD) as a negative control and overexpression of S6kCA (HH > S6kCA) as a positive control. Hemocytes from 200 larvae were pooled per genotype and 30 µg of protein was loaded into each well. In S6kCA, Threonine at T398 is replaced by glutamic acid rendering the site at least partially independent of upstream mTOR activation. ATP5 was used as a loading control. As evident from the blot, the S6k protein levels were very low in control hemocytes. The amount of S6k decreased to virtually undetectable levels in the S6k knock-down and increased in S6k overexpressing hemocytes, verifying that the antibody is detecting S6k in vivo. B) pS6k and S6k in hemocytes with mTorWT overexpression and in hemocytes from wasp-infected larvae. Even though we increased the pools of larvae to 300, and loaded 35 µg of protein into each well, the levels of pS6k were in general very low in all samples, again especially in the uninfected control hemocytes. A bulk larval hemocyte collection method would be required to have enough protein for proper detection of S6k protein and phospho-protein in untreated hemocyte samples; B’) Ratio of pS6k to total S6k in hemocytes. the ratio of the pS6k band intensity of three blots to one blot of the total S6k of the same samples. ns – not significant. The data are available in the S7 Table. (TIF) [file pgen.1012094.s019.tif]

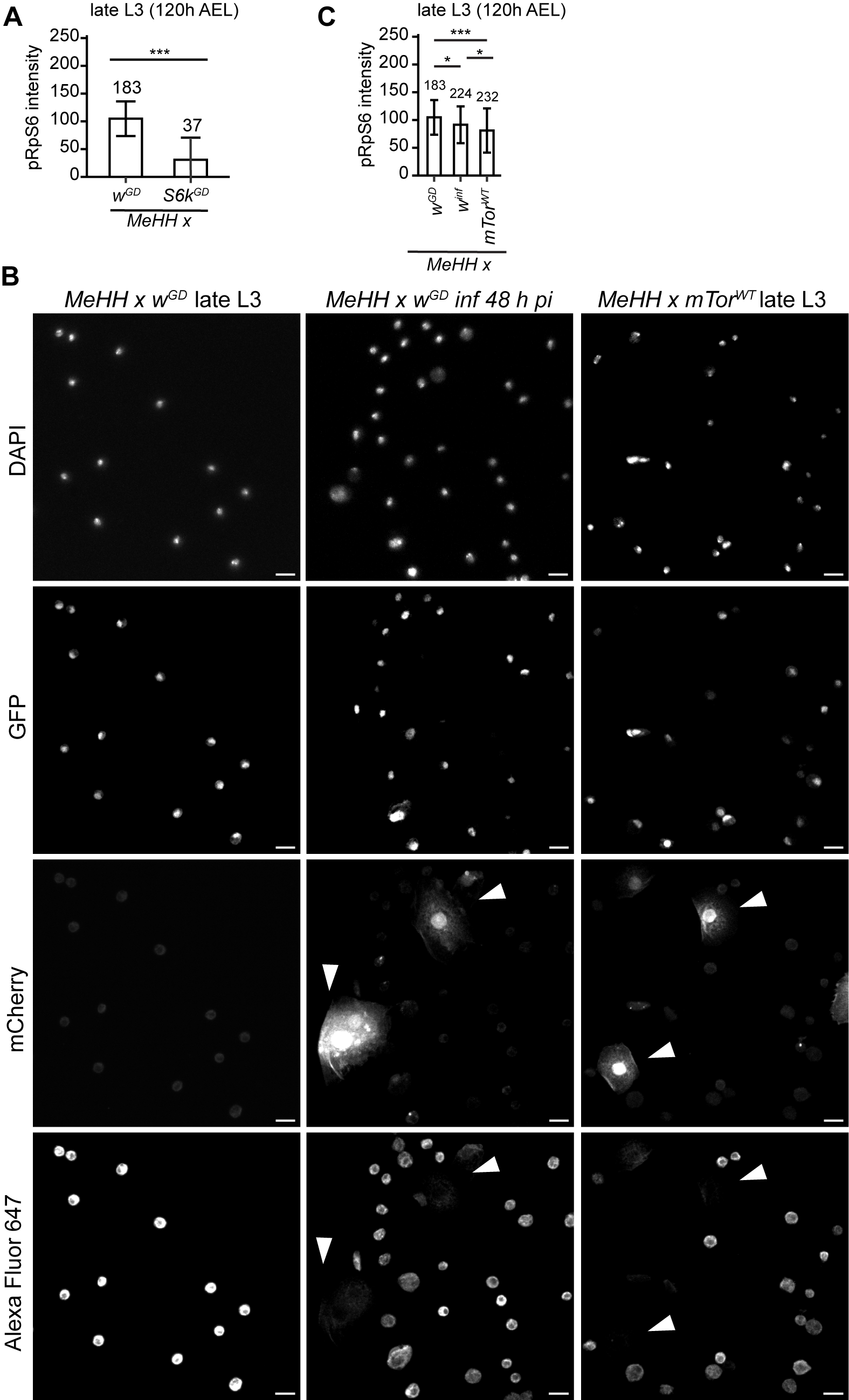

Supplement: S13 Fig — A) To verify the functionality of the pRps6 antibody in hemocytes, we knocked down S6k (HH > S6kGD) and checked the effect on the pRpS6 (Alexa Fluor 647) signal intensity. As expected, the pRpS6 staining intensity was reduced in S6k knock-down hemocytes. B) Example images of the pRpS6 staining in control (MeHH > wGD), wasp-infected (MeHH > wGD inf) and mTorWT overexpressing (MeHH > mTorWT) hemocytes. DAPI – nuclei, GFP – eaterGFP-positive hemocytes, mCherry – MsnCherry-positive hemocytes, Alexa Fluor 647 – pRpS6 staining. Arrowheads point to lamellocytes. The images here are the same as the maximum intensity projections in Fig 6A-C, but here all channels are shown separately. C) Quantification of the pRpS6 stain intensity in hemocytes from late third instar larvae. Significance levels: *** p < 0.0001, ** p < 0.001, * p < 0.05. The data are available in the S7 Table. (TIF) [file pgen.1012094.s020.tif]

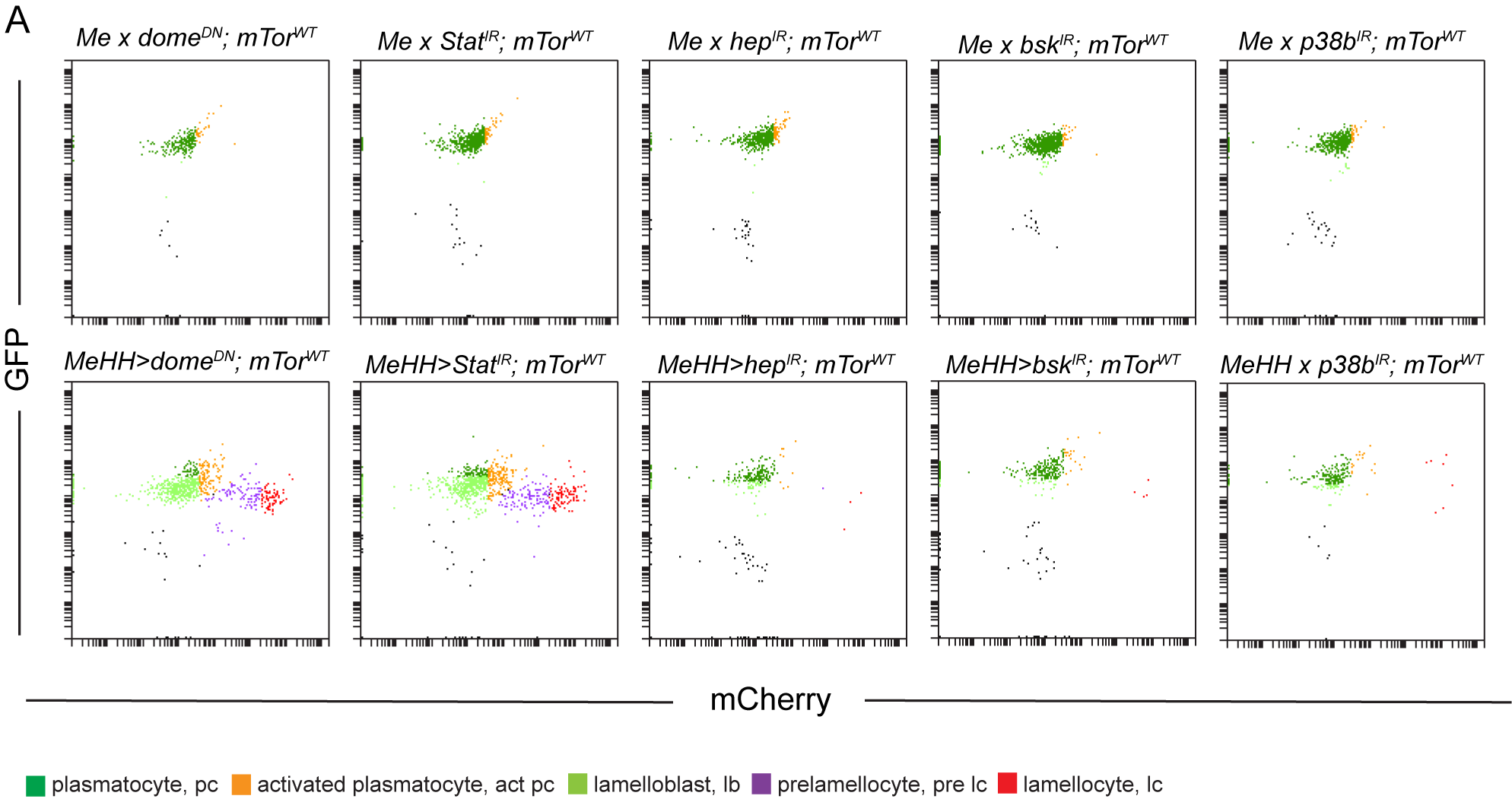

Supplement: S14 Fig — A) Me x mTorWTdomeDN and MeHH > mTorWTdomeDN. B) Me x mTorWT StatIR and MeHH > mTorWT StatIR. C) Me x mTorWT hepIR and MeHH > mTorWT hepIR; Me x mTorWT bskIR and MeHH > mTorWT bskIR. D) Me x mTorWT p38bIR and MeHH > mTorWT p38bIR. (TIF) [file pgen.1012094.s021.tif]

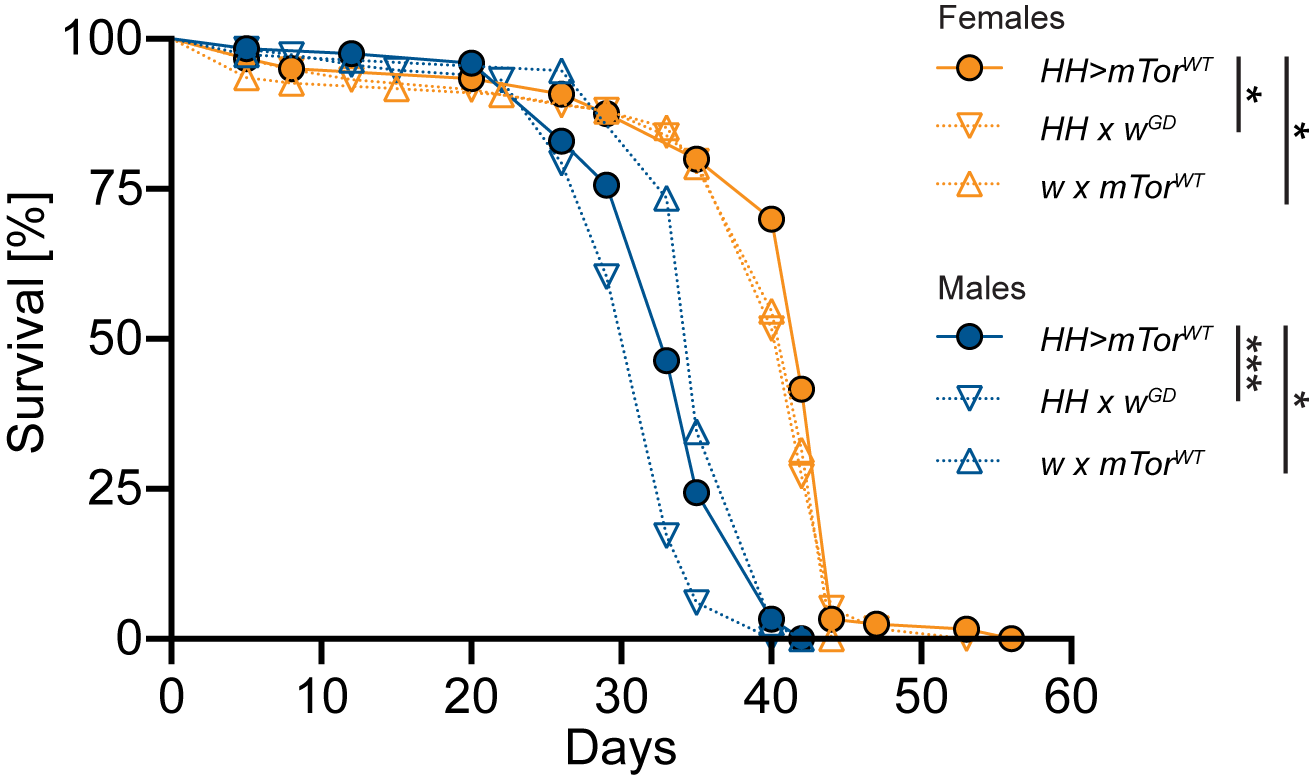

Supplement: S15 Fig — Lifespan of HH > wGD (118 females and 116 males), w x mTorWT (108 females and 103 males) and mTorWT-overexpressing (HH > mTorWT, 120 females and 120 males) flies. Flies were maintained at 29 °C. Significance levels: *** p < 0.0001, ** p < 0.001, * p < 0.05. The data are available in the S7 Table. (TIF) [file pgen.1012094.s022.tif]
