# Supplementary material for: mTOR signaling regulates demand-adapted hematopoiesis and metabolic reprogramming required for an effective cellular immune response in Drosophila melanogaster larvae
Source: PLoS Genet. 2026 Mar 24;22(3):e1012094. doi: 10.1371/journal.pgen.1012094 (PMC13038113; doi:10.1371/journal.pgen.1012094)
Supplement: Attachment — Submitted filename: Response to Reviewers.docx [file pgen.1012094.s024.docx]

We thank the reviewer #1 for the justified and insightful comments regarding our manuscript. Below, we address each point and remark in detail.

Reviewer #1: This study examines the role of mTOR in activating hemocytes to defend Drosophila larvae against parasitoids. This important study has been conducted thoroughly and carefully. The study is based on genetic manipulations specifically in hemocytes and analyzes the effects of these manipulations on hematopoiesis and gene expression in hemocytes. Similar to infection, mTOR overexpression leads to hemocyte activation and increased resistance, resulting in largely similar global changes in gene expression, including metabolic genes. Conversely, mTOR downregulation reduces activated plasmatocytes and resistance. This supports the authors' conclusion that mTOR is important for the metabolic switch in hemocytes that are activated to fight the parasitoid. However, analysis of expression after mTOR suppression and, in particular, direct analysis of metabolism is lacking so the results clearly show that mTOR plays an important role in hemocyte activation and immune response, and although it is clear that mTOR is a key metabolic regulator, the results of this work did not directly test the role of mTOR in the metabolic switch of hemocytes. This is my only major criticism of the work.

We agree with the reviewer. However, we wish to point out that our interest was to look into hemocyte activation and lamellocyte differentiation, induced either genetically (mTor overexpression) or naturally (by wasps). mTor suppression only reduced plasmatocytes in the uninfected larvae and activated plasmatocytes after wasp infection. Therefore, at the time, we anticipated that RNA seq on hemocytes with mTor suppression would not have yielded much new information on hemocyte activation and lamellocyte differentiation. This said, any future experiments on either gene expressional or metabolic changes should be studied in the context of *mTor* overexpression and suppression.

As part of the minor revision, I recommend toning down statements that draw strong conclusions about the effects on metabolism. In particular, I recommend emphasizing the limitations of this work.

First, RNAseq suggests many things, but it is still far from analyzing metabolism.

Second, I would emphasize more often that this is bulk RNAseq. The authors correctly emphasize this point in some passages. Therefore, conclusions about specific types of hemocytes are limited. The only analysis in this work that differentiates the effect on specific types of hemocytes is the analysis of S6k phosphorylation using an antibody directly in hemocytes.

We agree that RNA‑seq alone does not provide a complete picture of the metabolic changes, and that some of our conclusive statements were too strong. Also, bulk RNA sequencing poses it own challenges regarding detailed data interpretation. Accordingly, we have revised the manuscript by adjusting the statements and conclusions related to metabolic alterations (pages 16, 18, 19, 23, 25 and 29). We have also more clearly emphasized the limitations of bulk RNA sequencing (p. 11).

Particular notes:

Page 21: “The other NADPH-producing enzymes Phosphogluconate dehydrogenase (Pgd; Fig. 7A, D), Malic enzyme (Men) and Isocitrate dehydrogenase (Idh; Fig. 7A, D’’) were downregulated implying that the main source of NADPH in activated hemocytes is Zw as shown earlier by Kazek et al. 2024 (63).” The upregulation of Zw and the downregulation of Pgd gene expression demonstrate the limitations of analyzing metabolism solely by mRNA expression. Both enzymes are required to complete the oxidative pentose phosphate pathway, so the statement that Zw is the main source of NADPH is misleading.

We have revised the text and modified the misleading conclusion (p. 17, lines 395-397).

Page 29/30: “Thus, collectively, our data imply that mTORC1 suppression is a characteristic of lamellocyte differentiation and function.”

This is a limitation of the study. Is the suppression of mTORC1 in differentiated lamellocytes important for their function? This was not tested, and this limitation should be mentioned in the discussion with the proposal of potential future experiments, such as activating mTORC1 specifically in lamellocytes upon infection.

We modified this statement in the discussion (p. 26, lines 641-644).

Page 30: “The metabolic switch from mitochondrial OXPHOS to aerobic glycolysis observed in our data…” This is an example of overly strong phrasing. The actual metabolic switch was not observed in this work. This is an example of where the limitations of the study should be considered.

We agree with the reviewer, and have now removed the statement on the observation of the metabolic switch in our data, p. 26, line 637.

Page 31: “Kazek et al. (63) showed by 13C labeling of Gln during the early course of infection (16 hours pi + 6 h Gln 13C incubation)” Note: 13C-Gln was not used in the Kazek et al. study, but rather, glutamine was labeled in vivo from 13C-labeled glucose fed to the larvae. Glutamine metabolism was then traced based on its labeling pattern.

We thank the reviewer for pointing out this misleading wording. We have changed the wording (p. 27, rows 679-680).

Page 32: “Our data indicate H2O2 production by upregulation of Duox and its cofactor moladietz in activated hemocytes.” ROS production in activated hemocytes remains questionable. Although Duox and moladietz expression increases in this study, their expression in hemocytes is extremely low based on their CPMs compared to other genes (STable4) — a finding that has been reported repeatedly in other studies using RNAseq on hemocytes. This calls into question the importance of Duox for ROS production in hemocytes.

We thank the reviewer for this insightful comment. We agree that our conclusion is too strong, and further experiments are required. To address this point, we have revised the manuscript to clarify the low baseline expression of *Duox* and *moladietz*, and we have added a cautionary remark regarding their expression levels (p. 18). We have also expanded the discussion on this result (p. 28), and added on reference on function of Duox in larval hemocytes (Kinoshita et al., ref 119)

Supporting data files with the data used to produce the figures' plots should be provided and specified in the figure legends.

We thank the reviewer for pointing this out. We have added a Supplementary Table 7 with the data plotted in the figures, and specified the location of the data in the figure legends.
